# Evaluation of the hepatitis c virus screening program in the Marche Region (Italy): experience from the Pesaro–Urbino health authority

**DOI:** 10.1007/s10096-026-05430-7

**Published:** 2026-02-11

**Authors:** Raffaele La Porta, Antonio Vitiello, Annalisa Belli, Davide Sisti, Andrea Zovi, Francesca Bruscolini, Saverio Guerra, Sara Carbonari, Sara Di Benedetto, Tania Rodini, Simone Barocci, Stefano Amatori

**Affiliations:** 1 Department of Clinical Pathology, AST Pesaro-Urbino, Urbino, Italy; 2 Ministry of Health, Directorate-General for Health Prevention, Rome, Italy; 3 Department of Biomolecular Sciences, University of Urbino Carlo Bo, Urbino, Italy; 4 School of Advanced Studies, University of Camerino, Camerino, Italy

**Keywords:** HCV, Hepatitis c virus, anti-HCV antibodies, HCV-RNA, Screening program, HCV elimination

## Abstract

**Background:**

Hepatitis C virus (HCV) infection remains a global health challenge despite the availability of highly effective direct-acting antivirals. Early case identification through population screening is crucial to achieve the WHO target of HCV elimination. We conducted a population-based screening program in the Marche Region (Italy), focusing on the Local Health Authority of Pesaro–Urbino, to evaluate the feasibility and first-year outcomes of the national HCV screening campaign.

**Methods:**

Between August 2023 and July 2024, individuals born between 1969 and 1989 residing in the Pesaro–Urbino area were invited to undergo free HCV testing. Screening was performed using serological anti-HCV antibody assays, and positive cases were further evaluated with HCV-RNA testing to confirm active infection.

**Results:**

A total of 6,319 individuals (12.4% of the eligible population) participated in the screening program. Anti-HCV antibodies were detected in 64 participants (1.0%). Among them, 8 cases (0.13% of the total screened) showed detectable HCV-RNA, confirming ongoing infection.

**Conclusions:**

The prevalence of active HCV infection in this population was low, consistent with declining national and European trends. However, the program successfully identified untreated viremic individuals who can now access curative therapy. Our findings highlight both the importance and the challenges of implementing large-scale screening campaigns in low-prevalence settings, emphasizing the need for tailored strategies to maximize participation rates and achieve elimination goals.

## Introduction

Hepatitis C virus (HCV) infection represents a major global public health burden and remains one of the leading causes of chronic liver disease, cirrhosis, and hepatocellular carcinoma worldwide [1, 2]. In the absence of antiviral treatment, chronic HCV infection progresses to cirrhosis or hepatocellular carcinoma in approximately 20% of cases, with a substantial risk of liver failure and death [3]. Despite the severity of its long-term complications, HCV infection is often asymptomatic for decades, which contributes to its underdiagnosis. In most cases, individuals become aware of their infection only through targeted or opportunistic screening [4, 5].

Globally, an estimated 71 million people (1% of the world population) live with chronic HCV infection, and approximately 1.75 million new infections occur each year [6, 7]. Recognizing this global challenge, the World Health Organization (WHO) launched in 2016 a comprehensive strategy to eliminate viral hepatitis as a public health threat by 2030, setting ambitious targets: a 90% increase in diagnosis, a 65% reduction in HCV-related mortality, and widespread access to curative treatment [8].

In Italy, the estimated prevalence of chronic HCV infection is around 1% of the general population [9]. However, reliable nationwide epidemiological data remain limited, since most surveillance systems have historically focused on acute viral hepatitis cases [10–12], which provide only a partial estimate of the true infection burden. Studies conducted in specific cohorts, including blood donors and hospital-based populations, report an HCV-RNA positivity rate of approximately 1% [13, 14]. To align with WHO elimination goals, in 2019 the Italian government allocated €71.5 million to implement a nationwide HCV screening initiative targeting individuals born between 1969 and 1989, as well as people with substance-use disorders and incarcerated individuals [15]. Legislative measures—including Decree Law No. 162 of December 30, 2019, later enacted through the Decreto Milleproroghe (December 28, 2020) and formalized by the State-Regions Conference of December 17, 2020—laid the regulatory groundwork for the national campaign. The program was officially launched with the Ministerial Decree of May 14, 2021 [16].

The availability of direct-acting antivirals (DAAs) has revolutionized HCV therapy, allowing sustained virological response rates above 90%, with excellent tolerability and minimal side effects [17, 18]. As a result, nationwide efforts to identify untreated individuals have gained momentum, emphasizing the role of screening in achieving elimination. An effective HCV screening strategy aims to detect infections in individuals who are unaware of their status and may unknowingly contribute to ongoing transmission. Early identification facilitates timely treatment initiation, prevents disease progression, and interrupts viral spread within the community. However, the COVID-19 pandemic caused a substantial delay in the implementation of the national program: the regional allocation of funds was postponed until 2021, and the first large-scale screenings began only in 2022. By July 2023, the campaign promoted by the Ministry of Health and adopted by the Marche Region was launched across all local health authorities.

The present study reports the first-year results of the screening campaign conducted in the Pesaro–Urbino Health Authority (Azienda Sanitaria Territoriale, AST). The analysis revealed a low prevalence (0.13%) of active HCV infection among previously untested individuals, underscoring the importance of sustained efforts to optimize screening strategies, enhance population engagement, and monitor real-world program performance across regions.

## Materials and methods

### Study design and setting

This was a prospective, population-based screening study conducted in the Local Health Authority (Azienda Sanitaria Territoriale, AST) of Pesaro–Urbino, Marche Region (Central Italy). The study was carried out between August 2023 and July 2024 as part of the Italian national HCV screening program established by law in 2020.

### Screening centers

**Fig. 1:**
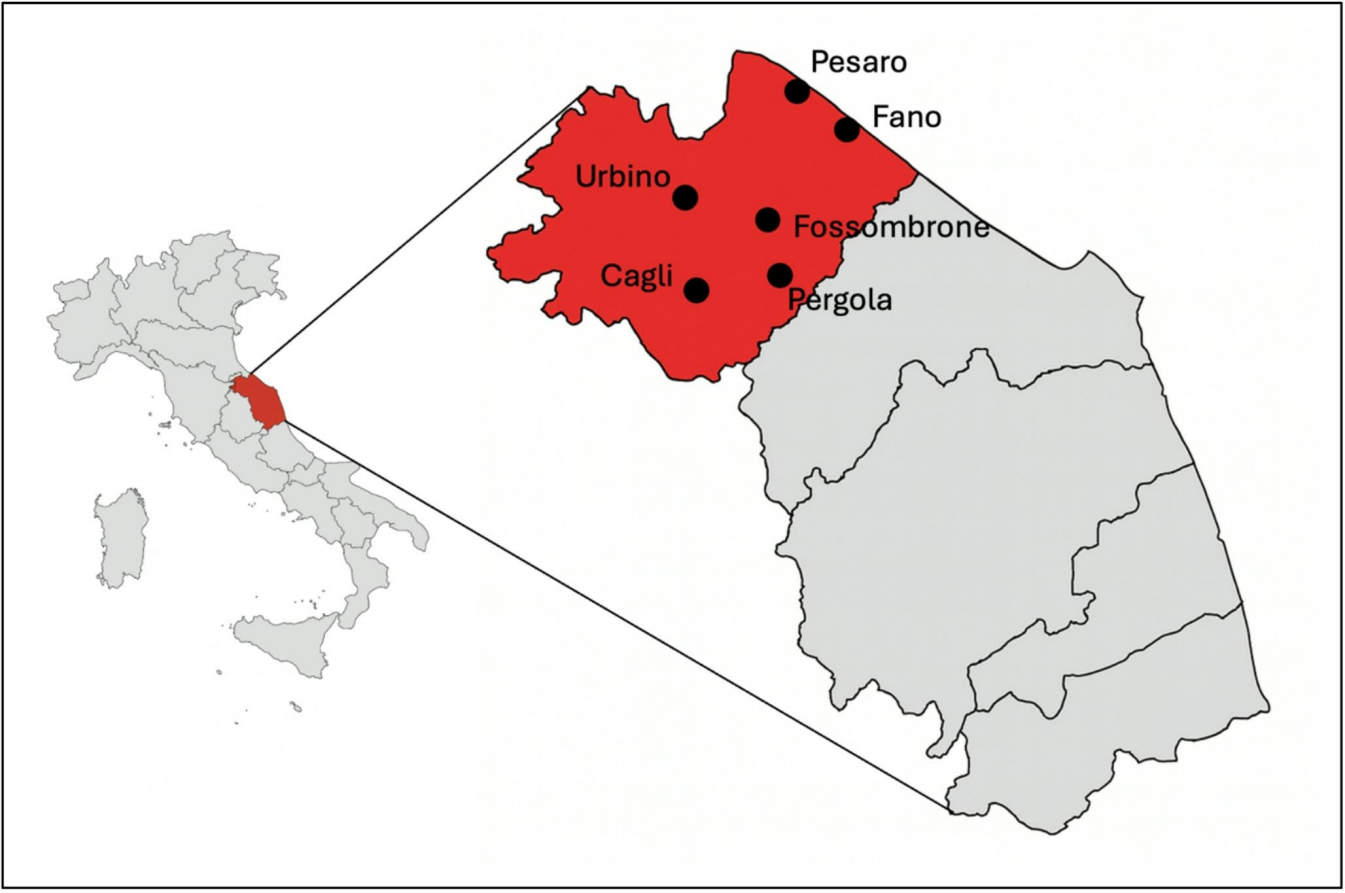
Geographic distribution of the screening centers included in the Pesaro–Urbino province. Map showing the location of the six blood collection centers participating in the HCV screening program

Six different centers were identified for the collection of anti-HCV samples (and consequently for the collection of HCV-RNA samples as well). The collection centers are reported in Fig. 1. These six collection centers are in different towns: Cagli (Ospedale di Comunità Celli), Fano (Presidio Ospedaliero Santa Croce), Fossombrone (Ospedale Civile), Pesaro (Presidio Ospedaliero San Salvatore), Pergola (Ospedale Santi Donnino e Carlo), and Urbino (Ospedale Santa Maria della Misericordia).

### Study population

Eligible participants were all residents born between 1969 and 1989 and registered in the AST Pesaro–Urbino catchment area. This cohort was identified according to the national screening policy targeting this birth cohort, with the rationale of intercepting undiagnosed infections in age groups not previously considered at risk [6, 15].

### Recruitment and data collection

Individuals were invited to participate through local health services, general practitioners, and public communication channels. Participation was voluntary and free of charge. At the time of testing, basic demographic information (age, sex, place of residence) was collected. Specific informed consent was provided authorizing the use of the clinical information, and participant data was fully anonymized through appropriate pseudonymization. Personal identifiers were removed from the dataset to ensure confidentiality. A total of 6,319 samples were collected.

### Screening procedures

For each participant, two blood samples were collected: the first tube was used for serological testing, and the second was reserved for molecular analysis when indicated. In cases of anti-HCV antibody positivity, HCV-RNA testing was performed to confirm active infection. Individuals with detectable HCV-RNA were subsequently referred to the regional specialist center for completion of the diagnostic work-up, including clinical evaluation and assessment of eligibility for antiviral therapy.

The screening program involved both hospital-based and community blood collection centers, to facilitate access and increase participation. All serum samples for anti-HCV testing were processed at the Hospital of Urbino, whereas HCV-RNA analyses were carried out at the Hospital of Pesaro (Italy). Anti-HCV antibody detection was performed using the LIAISON^®^ XL Murex HCV Ab assay (DiaSorin S.p.A., Saluggia, Italy), an indirect chemiluminescent immunoassay (CLIA) that employs two recombinant HCV antigens (core and NS4) coated on magnetic particles as solid phase, together with a biotinylated NS3 antigen in aqueous solution. The assay was run on the LIAISON^®^ XL analyzer according to the manufacturer’s instructions [19]. Quality control procedures were systematically performed prior to each analytical session.

For HCV-RNA detection and quantification, plasma samples were analyzed using a real-time polymerase chain reaction (PCR) assay on the Cobas^®^ 6800 System (Roche Diagnostics Ltd., Burgess Hill, UK). Cobas HCV Sterilin round-base polystyrene (LP4) tubes (Thermo Fisher Scientific, Hemel Hempstead, UK) or centrifuged BD Vacutainer^®^ EDTA whole-blood tubes (Becton Dickinson, Oxford, UK) were directly loaded into the sample supply module of the instrument.

All results were recorded in a dedicated Microsoft Excel database, with each entry fully anonymized prior to analysis [20]. Data were subsequently aggregated for statistical evaluation.

### Statistical analysis

Categorical variables were expressed as absolute frequencies and percentages, while continuous variables (age) were reported as mean ± standard deviation (SD). Comparisons between categorical variables were performed using the Chi-square (χ²) test. This test was applied to assess: (i) potential differences in the distribution of anti-HCV positive and negative results across the various collection centers; (ii) the distribution of sex across different age groups; and (iii) the distribution of anti-HCV outcomes (positive or negative) within the considered age strata. When the expected frequency in any cell was < 5, the Fisher’s exact test was employed instead of the standard Chi-square test to evaluate the distribution of HCV-RNA outcomes across collection centers.

To examine the distribution of positive cases relative to the population size of each municipality and, separately, to the population size within the target age range, a Chi-square goodness-of-fit test for proportions was performed. Cramer’s V was calculated as a measure of effect size to quantify the strength of the association.

In addition, considering the sensitivity (93.18%) and specificity (99.35%) of the screening test [21], and the observed prevalence of infection, the positive predictive value (PPV) was computed according to the formula:

$$\:PV+=\:\frac{Sens*Pr}{Sens*Pr+\left(1-Spec\right)*(1-Sens)\:}$$

Legend: Pr = Prevalence; Sens = Sensibility; Spec = Specificity.

All statistical tests were two-sided, and a p-value < 0.05 was considered statistically significant. Analyses were conducted using Microsoft Excel (Microsoft Corporation, Redmond, WA, USA) and R Studio (R Foundation for Statistical Computing, Vienna, Austria).

## Results

### Overview of the screened cohort

The HCV screening program was implemented across six blood collection centers within the AST Pesaro–Urbino network and was carried out between August 1, 2023, and July 31, 2024. A total of 6,319 individuals participated in the screening initiative, including 2,579 males (40.8%) and 3,740 females (59.2%). A chi-square test for independence revealed no statistically significant difference in the sex distribution across the different age groups, although the result approached significance (χ²_(3)_ = 7.29, *p* = 0.06). Nevertheless, inspection of expected versus observed frequencies indicated a consistently higher participation rate among women in all age strata (Table 1).

**Table 1:** Demographic characteristics of individuals participating in the HCV screening program

Total	M *n* (%)	F *n* (%)	Anti-HCV^+^ *n*	RNA-HCV^+^ *n*
	2,579 (40.8%)	3,740 (59.2%)	64	8
1969–1974	1,232 (47.8%)	1,671 (44.7%)	32	*5*
1975–1979	625 (24.2%)	932 (24.9%)	2	0
1980–1984	417 (16.17%)	681 (182%)	9	3
1985–1989	305 (11.8%)	456 (12.1%)	3	0

Regarding anti-HCV seropositivity, 64 individuals (1.01%) tested positive. Among these, 56.3% were males and 43.7% females, indicating a significantly higher prevalence in men (Chi² = 6.376; *p* < 0.05), although with a small effect size (Table 2).

**Table 2:** Gender-specific distribution of anti-HCV positivity

Total	Anti-HCV^+^ *n* (%)	RNA-HCV^+^ *n* (%)
	64	8
Males	36 (56.3%)	5 (62.5%)
Females	28 (43.7%)	3 (37.5%)

**Fig. 2:**
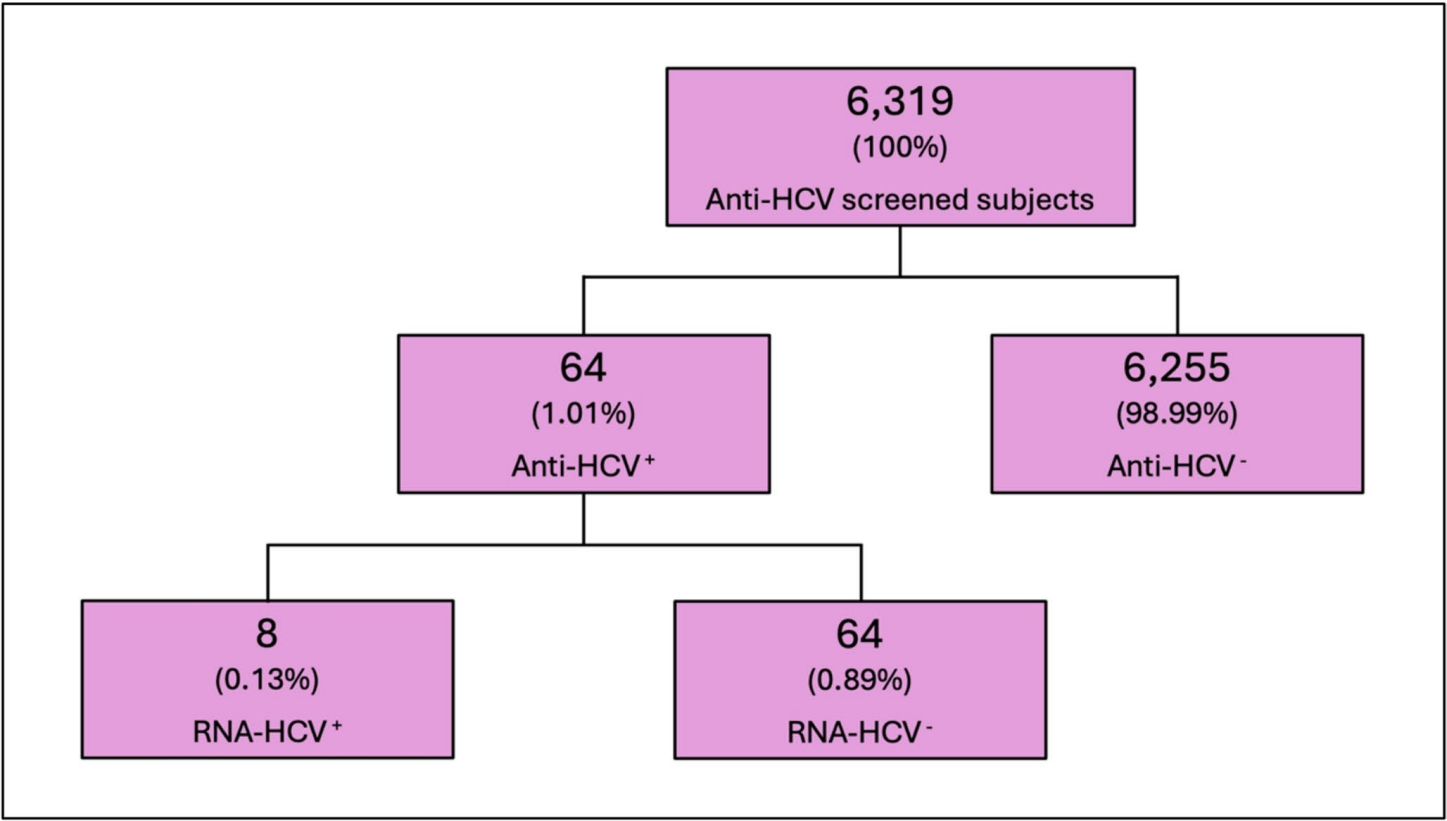
Flow diagram of HCV screening outcomes. Overview of the screening cascade showing the total number of individuals tested, anti-HCV positive cases, and subjects with confirmed HCV-RNA positivity

The screening cascade is summarized in Fig. 2, which illustrates the sequential flow from initial serological testing to molecular confirmation. Among the 64 anti-HCV positive cases, 8 individuals (12.5%) were confirmed HCV-RNA positive, corresponding to a prevalence of active infection of 0.13% in the screened population.

**Fig. 3:**
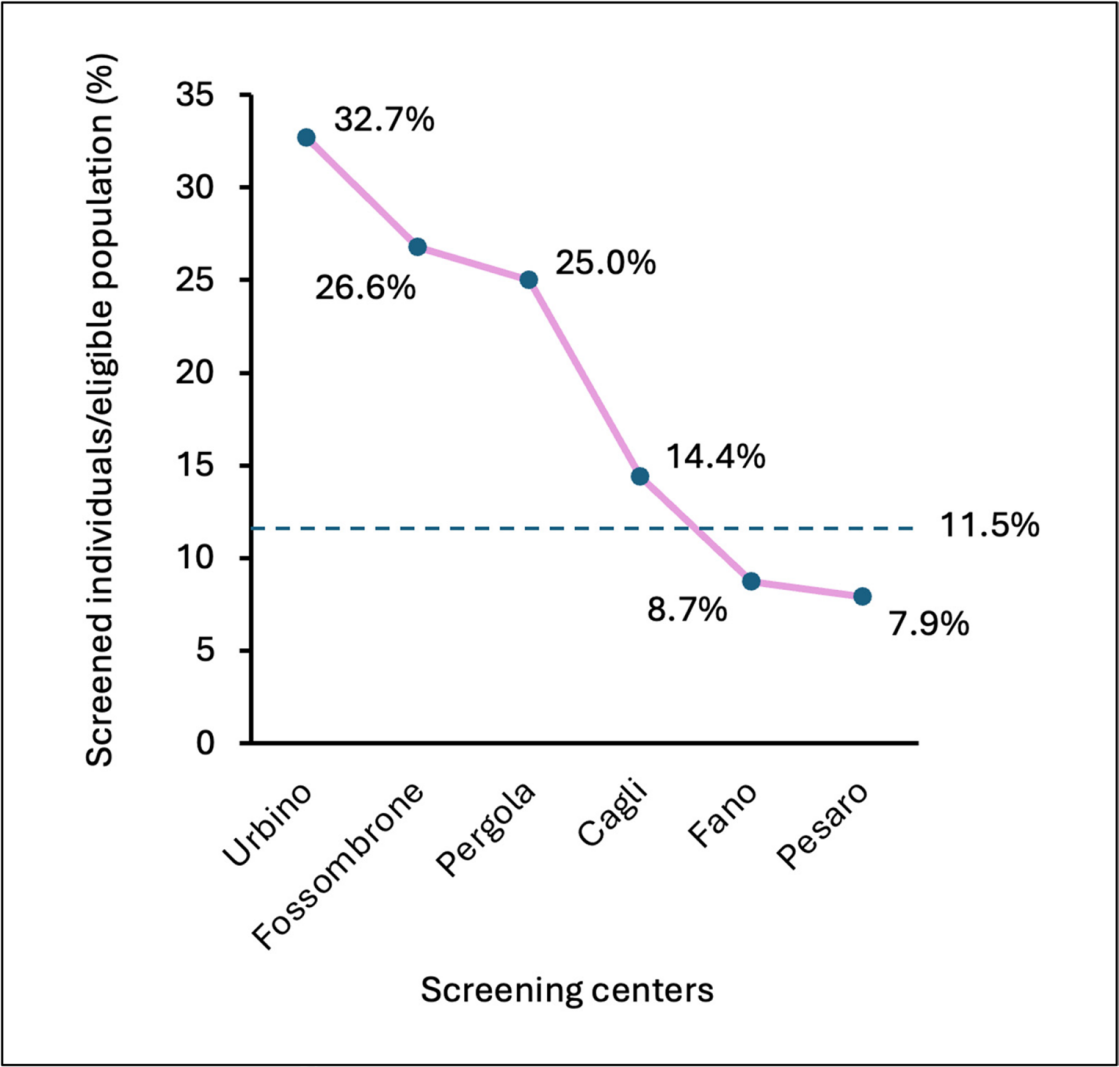
Proportion of screened individuals relative to the eligible population (birth years 1969–1989). The figure shows the proportion of participants who underwent anti-HCV screening in each municipality, calculated as the ratio between the number of screened individuals and the total population within the target age range. The dashed horizontal line represents the overall mean screening rate across all municipalities (*p* < 0.05)

To assess whether adherence to the screening program varied geographically, a Chi-square goodness-of-fit test was applied to the proportions of participants from each municipality relative to the eligible population size. The analysis revealed a highly significant difference (*p* < 0.001), with a Cramer’s V = 0.65, indicating a large effect size. This result demonstrates that participation was non-uniform across municipalities, with marked differences in adherence rates between catchment areas (Fig. 3).

### Screening program results

Among the 54,828 residents born between 1969 and 1989 in the six municipalities included in the program, 6,319 individuals underwent HCV testing, corresponding to an overall adherence rate of 11.52%. Of the 6,319 participants, 64 subjects (1.01%) tested positive for anti-HCV antibodies (Tables 1, 2 and 3). Although a higher proportion of women participated in the screening (59.2% vs. 40.8%), anti-HCV seropositivity was more frequent among men (56.3% male vs. 43.7% female). Statistical analysis revealed no significant association between sex and anti-HCV positivity (χ²_(5)_ = 2.50, *p* = 0.77; Cramer’s V = 0.02). Likewise, the distribution of positive cases across different age groups was not statistically significant (χ²_(3)_ = 4.69, *p* = 0.19; Cramer’s V = 0.03).

**Table 3:** Distribution of screening tests and positive outcomes across collection centers. *Percentages of anti-HCV–positive and HCV-RNA–positive cases are calculated relative to the total number of tests performed in each center

Overall population	Total	Cagli	Fano	Fossombrone	Pesaro	Pergola	Urbino
	192,172	7,951	59,963	9,077	95,580	5,762	13,839
1969–1989 population	54,828	2,212	17,522	2,606	27,097	1,549	3,842
Screening tests (n)	6,319	319	115	693	2,149	388	1,255
Positive anti-HCV (PPV) n (%*)	64 (1%)	4 (1.2%)	12 (0.8%)	10 (1.4%)	23 (1.1%)	4 (1.0%)	11 (0.9%)
Positive HCV-RNA n (%*)	8 (0.127%)	0 (0%)	0 (0%)	1 (0.016%)	4 (0.063%)	2 (0.032%)	1 (0.016%)

Among the 64 individuals who were anti-HCV positive, 8 subjects (12.5%)—5 men and 3 women—were confirmed as HCV-RNA positive, indicating active viral infection. Given the limited number of RNA-positive cases, a Fisher’s exact test was applied to assess the distribution of viremia across screening centers, revealing no statistically significant differences (*p* = 0.20).

The overall prevalence of active HCV infection in the screened population was therefore 0.13%. Based on the known sensitivity and specificity of the assay employed, the positive predictive value (PPV) and negative predictive value (NPV) of the screening test were calculated. The resulting PPV was 0.157, indicating that approximately 15.7% of seropositive individuals were confirmed to have active infection, while the NPV was 0.999, confirming that virtually all individuals testing negative were truly uninfected.

## Discussion

The World Health Organization (WHO) global initiative to eradicate hepatitis C virus (HCV) infection has prompted the development of national and regional screening strategies aimed at identifying and managing infected individuals. Laboratory-based diagnostic testing remains a cornerstone of these strategies, playing a critical role in detecting HCV-reactive cases, interrupting transmission, and reducing both infection rates and hepatitis-related mortality [22]. The advent of direct-acting antivirals (DAAs) has profoundly transformed HCV management, achieving cure rates exceeding 90% and dramatically improving long-term clinical outcomes [23, 24]. However, such therapeutic success depends on timely diagnosis, which can only be achieved through effective screening programs. The present study therefore contributes to the ongoing implementation of targeted, evidence-based screening strategies, which could also serve as a model for other viral infections [25].

Despite the strengths, several limitations must be acknowledged. The first limitation concerns the sample size, as the present report represents only an interim analysis of a broader regional opportunistic screening initiative that is still ongoing and will progressively include additional centers in the coming months. A second limitation is the age restriction of the enrolled population (birth years 1969–1989), which limits the comparability of our findings with studies including broader age cohorts. This selection criterion was defined by the Italian Ministry of Health, based on analyses demonstrating the cost-effectiveness of focusing on this birth cohort compared with either universal or high-risk-only screening models. This strategy was shown to provide the optimal balance between epidemiological yield and resource allocation, given that a significant proportion of undiagnosed infections persists within this age group and may otherwise remain silent for years [26]. Another limitation lies in the limited demographic dataset, as only sex and age were collected for each participant; therefore, data on country of origin or migrant status were not available, precluding an evaluation of the contribution of first-generation migrant populations from high-prevalence regions to the observed HCV burden. Moreover, information on previous awareness of HCV serostatus was not systematically recorded. Although the screening program was designed to primarily target individuals presumed to be unaware of their infection status, it was not possible to formally distinguish newly diagnosed anti-HCV–positive individuals from subjects with a known prior diagnosis who were re-evaluated within the program. Furthermore, although all subjects with confirmed HCV-RNA positivity were referred to specialized centers for further clinical management, complete data on linkage to care and potential loss to follow-up were not yet available at the time of this interim analysis. Finally, detailed clinical information, including liver disease stage and degree of hepatic fibrosis among viremic individuals, was not accessible, as these assessments are performed at referral centers and will be integrated in future analyses. Nonetheless, the current findings provide valuable epidemiological insight into the local prevalence of HCV infection within the Pesaro–Urbino Health Authority (AST), representing a meaningful contribution to the regional understanding of HCV epidemiology. Preliminary results from this study were previously published [27]. The present work represents an updated, corrected, and extended version based on a refined interpretation of the findings.

Our data reveal that active HCV infection (HCV-RNA positivity) is markedly lower than previously reported in national studies [28, 29]. The present study summarizes the outcomes of the first year of the regional screening campaign conducted in the AST Pesaro–Urbino (Marche Region), focusing on individuals unaware of their infection status—the so-called “shadow population.” This initiative represents the first investigation of its kind in the Marche Region, and includes data collected from individuals born between 1969 and 1989. The observed prevalence of active infection (0.13%) aligns with recent findings from northern Italy (Lombardy: 0.1%) [30]. Previous studies targeting the same 1969–1989 birth cohort reported comparable rates of active HCV infection—0.05% and 0.07%, respectively [31, 32]. In contrast, investigations including participants of all ages have found higher prevalence estimates, ranging from 0.5% to 0.7% among hospitalized patients [33, 34] and 0.07% among individuals tested for anti–SARS-CoV-2 antibodies or vaccination [35]. Collectively, these recent studies demonstrate a substantial decline in HCV prevalence compared with pre-2019 data [36], likely reflecting the widespread use and success of DAA-based antiviral therapies in recent years.

Our findings further confirm that HCV prevalence is very low among individuals born between 1969 and 1989, consistent with previous Italian studies [37]. The higher prevalence observed among males and older age groups mirrors trends described in other national and regional analyses, suggesting that future screening efforts should prioritize these subpopulations. Evidence from Spain further supports this approach. The CRIVALVIR-FOCUS opportunistic screening program reported the highest HCV prevalence in individuals aged 45–64 years, with most infections detected in subjects previously unaware of their status. Similarly, the second Spanish national seroprevalence study confirmed that residual HCV burden is concentrated in older age groups, particularly among men and individuals born outside Spain [38, 39]. Accordingly, extending the upper age limit for HCV screening appears a plausible and potentially high-yield strategy for identifying residual reservoirs of infection. Of note, a large proportion of anti-HCV–positive individuals were HCV-RNA negative (87.5%), resulting in a relatively low rate of active infection among seropositive subjects. This finding is consistent with recent Italian and European screening studies conducted in the DAA era and may reflect, at least in part, the widespread availability of highly effective antiviral therapies, together with spontaneous viral clearance, which have been associated with a reduction in the prevalence of active infection despite persistent anti-HCV seropositivity. Similar proportions of antibody-positive but RNA-negative individuals have been reported in other opportunistic screening programs in low-prevalence settings [33].

From a public health perspective, the prevention of HCV infection requires reducing exposure risks among populations most vulnerable to transmission. Consequently, continuous awareness campaigns and screening programs are essential for identifying individuals unaware of their infection and linking them to care. In the absence of an effective vaccine, serological and molecular screening remains the primary strategy for controlling HCV spread. Strengthened collaboration among healthcare providers, researchers, and policymakers is therefore critical to refine existing protocols and advance toward the eradication of HCV. The relatively low prevalence of 0.13% observed in this study is unlikely to be explained by selection bias, as the screening was offered to all individuals accessing healthcare services within the target age range. It is, however, plausible that prevalence would have been even lower if the screening had included the entire general population, including individuals who rarely interact with healthcare facilities [40].

Evidence from Italy supports the cost-effectiveness of HCV screening and DAA treatment from the perspective of the national health system (SSN), reinforcing the rationale for sustained investment in elimination strategies. Treating infections identified through screening has been demonstrated to yield significant health and economic benefits, reducing the burden of HCV-related liver disease and its extrahepatic comorbidities. Early viral eradication can substantially mitigate these outcomes, confirming that screening is not only a fundamental component of HCV elimination policy but also economically advantageous [26]. The active offer of screening thus represents an important milestone, requiring coordinated regional governance capable of integrating community and hospital care pathways through well-organized interdisciplinary collaboration. Each region must define clear objectives and strategies to consolidate current achievements and ensure future progress toward the 2030 HCV elimination goal.

Finally, it is worth reiterating that this report presents only the first interim analysis of an ongoing regional program that will progressively include other centers across the Marche Region. The continued expansion of such initiatives at both regional and national levels will facilitate the identification of previously undiagnosed HCV-positive individuals, improve the cascade of care, and accelerate progress toward the WHO target of HCV elimination by 2030. Integrating these findings within a broader epidemiological framework will support the refinement of regional and national strategies, ensuring that screening efforts are efficiently directed toward the populations at greatest risk.

## Data Availability

Not Applicable.

## Abbreviations

AST: Azienda Sanitaria Territoriale
COVID-19: coronavirus disease-19
DAA: direct-acting antivirals
HCV: hepatitis C virus
SD: standard deviation
WHO: World Health Organization
